# Supplementary material for: Comparison of the Quality of Life of Patients with Breast or Colon Cancer with an Arm Vein Port (TIVAD) Versus a Peripherally Inserted Central Catheter (PICC)
Source: Curr Oncol. 2021 Apr 9;28(2):1495–506. doi: 10.3390/curroncol28020141 (PMC8167661; doi:10.3390/curroncol28020141)
Supplement: Supplementary file 1 [file curroncol-28-00141-s001.pdf]

Supplement

Table S1. Quality of Life Questionnaires related to the venous access device (QLVAD).

Satisfaction and Quality of Life Survey	Classification
1- Are you right or left handed?	Procedural
Right or Left	
2- Arm used for device implantation?	Procedural
Right or Left	
3 - Did you choose which arm you wanted the device implanted in?	Procedural
Yes	
No	
If No, please explain why, if possible.	
Text	
4 - Do you find it easy to present your device for treatment or blood sampling?	Treatment
Yes	
No	
If No, what issues do you encounter?	
Text	
5 - Please tell us how painful it was for you to have your device implanted. (Circle a number)	Procedural
1 2 3 4 5 6 7 8 9 10	
Minimal Moderate Severe	
6 - Do you feel that your device is too visible?	Psychosocial
Yes	
No	
7 - Do you feel that your device is unsightly or ugly?	Psychosocial
Yes	
No	
8 - Do you feel that you have changed the way you dress due to your device?	Psychosocial
Yes	
No	
9 - Have people commented on your device when they see it?	Psychosocial
Yes	
No	
10 - Do you try and cover your device with clothing?	Psychosocial
Yes	
No	
11 - Does your device make you feel anxious (worried)?	Psychosocial
Yes	

No	
12 - Does your device make you feel angry?	Psychosocial
Yes	
No	
13- Does your device make you feel self-conscious?	Psychosocial
Yes	
No	
14 - Does your device remind you of your illness?	Psychosocial
Yes	
No	
15 - Does your device make you feel embarrassed?	Psychosocial
Yes	
No	
16 - Are you worried that your device might become damaged?	Treatment
Yes	
No	
17 - Are you worried that your device might become blocked?	Treatment
Yes	
No	
18 - Are you worried that your device might become infected?	Treatment
Yes	
No	
19 - Does your device bother you when performing your work related activities?	Psychosocial
Yes	
No	
If Yes, please explain how.	
Text	
20 - Does your device bother you when you shower, bath, or perform personal hygiene?	Psychosocial
Yes	
No	
If Yes, please explain how.	
Text	
21 - Does your device bother you when you engage in sports or exercise?	Psychosocial
Yes	
No	
If Yes, please explain how.	
Text	
22 - Does your device bother you during social activities?	Psychosocial

Yes	
No	
If Yes, please explain how.	
Text	
23 - Does your device bother you when you are lying down in bed?	Psychosocial
Yes	
No	
If Yes, please explain how.	
Text	
24 - Does your device hurt?	Treatment
Yes	
No	
If Yes, please explain how.	
Text	
25 - Rate the degree of discomfort you experience during needle insertion into the device. (Circle a number)	Treatment
1 2 3 4 5 6 7 8 9 10	
Minimal Moderate Severe	
26 - Rate the degree of discomfort you experience during treatment infusion. (Circle a number)	Treatment
1 2 3 4 5 6 7 8 9 10	
Minimal Moderate Severe	
27 - Rate the degree of discomfort you experience during needle removal after treatment. (Circle a number).	Treatment
1 2 3 4 5 6 7 8 9 10	
Minimal Moderate Severe	
28 - Do you believe that insertion of your device was a good thing to have done? (Circle a number)	Psychosocial
1 2 3 4 5 6 7 8 9 10	
No Probably Definitely	
29 - Assess your degree of satisfaction with your device. (Circle a number)	Psychosocial
1 2 3 4 5 6 7 8 9 10	
Not Moderately Very	
30 - If you had to have a device implanted for another session of treatment during your life, would you have another	Psychosocial
1 2 3 4 5 6 7 8 9 10	
No Probably Definitely	

Table S2. Mixed effects model showing QOL of patients with a port versus a PICC.

Outcome of Interest*	Covariate	Estimate	95% CI	P values
Procedural scores	Intercept	4.87	4.10 – 5.73	<0.0001
	Type of device			
	Port	Ref		
	PICC	-1.98	-0.92 – (-3.05)	<0.001
	Survey			
	1 month	Ref		

	3 month	0.11	-0.47 – 0.69	0.713
Psychosocial scores	Intercept	3.38	2.32 to 4.43	<0.0001
	Type of device			
	Port	Ref		
	PICC	2.18	0.83 – 3.53	0.002
	Survey			
	1 month	Ref		
	3 month	-0.13	-0.58 – 0.84	0.71
Treatment scores	Intercept	2.79	2.12 – 3.45	<0.0001
	Type of device			
	Port	Ref		
	PICC	-0.36	-1.21 – 0.49	0.40
	Survey			
	1 month	Ref		
	3 month	-0.28	-0.77 – 0.22	0.27
Treatment scores	Intercept	2.79	2.12 – 3.45	<0.0001
	Type of device			
	Port	Ref		
	PICC	-0.36	-1.21 – 0.49	0.40
	Survey			
	1 month	Ref		
	3 month	-0.28	-0.77 – 0.22	0.27

*Satisfaction score was not significant and is not reported.

Table S3. PACS Detected Complications Related to Arm PICCs and Port.

**Complications**	**PICC N=50**	**Port N=51**	**P-values**
	n (%) [days after device insertion]	n (%) [days after device insertion]	
Infection (Septicemia)	0 (0%)	1(2%) [64]	0.999
Phlebitis	3 (6%) [8,9,13]	2 (4%) [15,27]	0.67
Device Occlusion	3 (6%) [60,96,205]	1 (2%) [488]	0.36
Arm Swelling	4 (8%) [12, 22, 33, 36]	4 (8%) [14,56,10,13]	0.999
Arm Pain	2 (4%) [33, 36]	1 (2%) [10]	0.61
Non-occlusive US detected Venous Thrombosis	3 (6%) [9, 33, 128]	3*(6%) [56, 15, 10]	0.999
Occlusive US detected Venous Thrombosis	4 (8%) [8,12, 13, 36]	6**(12%) [10, 13,14, 15, 27, 56]	0.74
Explantation – non-routine	0 (0%)	3 (6%) [15, 34, 64]	0.24
Wound dehiscence	0 (0%)	1(2%) [34]	0.999
Total Complications	19 (38%)	21(41%)	0.83

*all were related to Bard port; **4/6 were related to Bard port.
